# Causal associations between chronic hepatitis B and COVID-19 in East Asian populations

**DOI:** 10.1186/s12985-023-02081-4

**Published:** 2023-06-01

**Authors:** Zhenguo Liu, Linnan Song, Junling Chen, Yongjun Zhou, Yuhao Wang, Libo Tang, Yongyin Li

**Affiliations:** State Key Laboratory of Organ Failure Research, Guangdong Provincial Key Laboratory of Viral Hepatitis Research, Department of Infectious Diseases, Nanfang Hospital, Southern Medical University, No. 1838 North Guangzhou Avenue, Guangzhou, 510515 China

**Keywords:** Chronic hepatitis B (CHB), Coronavirus disease 2019 (COVID-19), Causal effect, Mendelian randomization (MR)

## Abstract

**Background:**

The relationship between chronic hepatitis B (CHB) and Coronavirus disease 2019 (COVID-19) has been inconsistent in traditional observational studies.

**Methods:**

We explored the total causal and direct causal associations between CHB and the three COVID-19 outcomes using univariate and multivariate Mendelian randomization (MR) analyses, respectively. Genome-wide association study datasets for CHB and COVID-19 were obtained from the Japan Biobank and the COVID-19 Host Genetics Initiative, respectively.

**Results:**

Univariate MR analysis showed that CHB increased the risk of SARS-CoV-2 infection (OR = 1.04, 95% CI 1.01–1.07, *P* = 3.39E−03), hospitalized COVID-19 (OR = 1.10, 95% CI 1.06–1.13, *P* = 7.31E−08), and severe COVID-19 (OR = 1.16, 95%CI 1.08–1.26, *P* = 1.43E−04). A series of subsequent sensitivity analyses ensured the stability and reliability of these results. In multivariable MR analyses adjusting for type 2 diabetes, body mass index, basophil count, and smoking, genetically related CHB is still positively associated with increased risk of SARS-CoV-2 infection (OR = 1.06, 95% CI 1.02–1.11, *P* = 1.44E−03) and hospitalized COVID-19 (OR = 1.12, 95% CI 1.07–1.16, *P* = 5.13E−07). However, the causal link between CHB and severe COVID-19 was attenuated after adjustment for the above variables. In addition, the MR analysis did not support the causal effect of COVID-19 on CHB.

**Conclusions:**

This study provides evidence that CHB increases COVID-19 susceptibility and severity among individuals of East Asian ancestry.

**Supplementary Information:**

The online version contains supplementary material available at 10.1186/s12985-023-02081-4.

## Introduction

The ongoing Coronavirus disease 2019 (COVID-19) pandemic, which is caused by the severe acute respiratory syndrome coronavirus 2 (SARS-CoV-2) virus, continues to pose a significant public health threat on a global scale [1, 2]. The disease can manifest with a broad spectrum of symptoms and severity levels [3]. Although the majority of cases are mild or asymptomatic, a subset of patients might progress to develop septic shock or acute respiratory distress syndrome, even experiencing mortality [4, 5]. Efforts to identify the underlying diseases or contributing factors for COVID-19, especially the severity, could facilitate the optimization of treatment and management strategies for COVID-19. Previous studies have established various risk factors associated with hospitalization or severe COVID-19, including advanced age [6], smoking [7], basophil count[8], and specific comorbidities like high body mass index (BMI) [9, 10] and type 2 diabetes [11].

Chronic hepatitis B (CHB) caused by the Hepatitis B virus (HBV) has imposed a sustained economic burden worldwide, especially in low- and middle-income regions or countries [12]. Until now, it is unclear whether CHB increases susceptibility or severity of COVID-19. Some clinical studies have reported that co-infection with HBV and SARS-CoV-2 may lead to elevated cytokine levels and transaminase levels [13–15]. However, several clinical studies have suggested that the extent of liver injury in patients with SARS-CoV-2 infection is not significantly different, regardless of whether they are co-infected with HBV or not [16–19]. Remarkably, a retrospective study conducted in Hong Kong revealed that neither past nor current HBV infection increased the risk of adverse outcomes or hepatic injury in patients with SARS-CoV-2 infection [20]. According to several studies, SARS-CoV-2-positive CHB patients had a higher risk of severe illness and fatality [21, 22]. The variability in these findings could be attributed to potential confounding factors, population heterogeneity, and relatively small sample sizes.

Mendelian randomization (MR) analysis uses genetic variants as instrumental variables to assess potential causal links between exposures and outcomes [23]. This method is not susceptible to confounding factors and is not prone to reverse causality because genetic variants are randomly allocated before birth [24]. However, there are no studies on the MR analysis between CHB and COVID-19 to date.

To investigate potential causal links between genetic susceptibility to CHB and three COVID-19 outcomes, namely SARS-CoV-2 infection, hospitalized COVID-19, and severe COVID-19, we conducted MR analyses using available genome-wide association study (GWAS) datasets. The causal effect of COVID-19 on CHB was also assessed.

## Research design and methods

### Study design

We first assessed the causal associations between CHB and the three COVID-19 outcomes by univariate MR analysis. This analysis method effectively emulates a randomized controlled trial by randomly assigning single nucleotide polymorphisms (SNPs) to offspring. The instrumental variable used in this MR analysis needs to meet three important conditions: (1) the SNPs must be strongly associated with exposure; (2) the SNPs are independent of potential confounders; (3) the genetic variant(s) only influence the outcome through risk factors [25]. Furthermore, the direct effect of genetically related CHB on three COVID-19 outcomes was investigated using multivariate MR (MVMR) analysis. We adjusted for known risk factors of COVID-19, including smoking behavior, body mass index, basophil count, and type 2 diabetes. The direct effect refers to the direct causal effect between exposure and outcome, excluding the mediating effect of other risk factors.

### GWAS and genetic instrumental variables for CHB

Summary statistics of CHB were from Ishigaki et al. [26], consisting of 1394 cases and 211,059 controls primarily from the BioBank Japan (BBJ) Project. Age, sex, and the top 5 principal components were adjusted. The BBJ program collects DNA and serum samples from 12 collaborating medical institutions in Japan and has enrolled approximately 200,000 patients of East Asian ancestry [27]. The 55 independent SNPs strongly associated with CHB were obtained based on the following criteria: (1) SNPs at the genome-wide significance level (*P* < 5 × 10^–8^); (2) SNPs clumping using the PLINK algorithm (linkage disequilibrium r^2^ < 0.01, within 100-kb distance). We conducted the clumping procedure using the East Asian reference panel from the 1000 Genomes Project. The intensity of each instrumental variant (IV) was assessed by an F-statistic calculated using the following formula: *F-statistics* = *Beta*^*2*^_*exposure*_*/SE*^*2*^_*exposure*_. Details about these instrumental variables are in Additional file 1: Table S1.

### GWAS data sets for three COVID-19 outcomes

We obtained summary statistics for COVID-19 outcomes from the COVID-19 Host Genetics Initiative GWAS meta-analysis round 7, which included individuals of East Asian ancestry [28]. The datasets consisted of SARS-CoV-2 infection (4459 cases and 36,121 controls), hospitalized COVID-19 (2882 cases and 31,200 controls), and severe COVID-19 (794 cases and 4862 controls). The dataset on SARS-CoV-2 infection primarily reflects the general susceptibility to the virus, while the datasets on hospitalized and severe COVID-19 mainly represent the disease's severity.

### MR analysis

Four different methods were used for MR analysis, including multiplicative random effects (IVW-MRE) and fixed effects inverse variance weighting (IVW-FE), MR-Egger regression, weighted median (WM) method, and weighted mode method. The IVW method provides robust and unbiased estimates in the absence of directional pleiotropy and heterogeneity. Although MR-Egger is tolerant to horizontal pleiotropy, it is sensitive to violating the Instrument Strength Independent of Direct Effect assumption and outliers. WM and weighted mode methods are generally less efficient but robust to outliers. In each MR analysis, we excluded SNPs that were absent from the outcome dataset, as well as palindromic SNPs with intermediate allele frequencies. The CHB and COVID-19 data sets were harmonized by aligning effect alleles.

To ensure that the results were stable and reliable, we conducted several sensitivity analyses. We used the Cochran Q statistic to quantify and test heterogeneity in the IVW method. To assess directional pleiotropy, we employed the MR-Egger intercept test, which detects horizontal pleiotropy when the intercept deviates from zero or has a *P* value ≤ 0.05. Leave-one-out (LOO) and funnel plot analyses were conducted to identify any single SNP that might be driving the causal estimates. In addition, the MR-PRESSO method was used to detect horizontal pleiotropy with a global test and to identify potential outlier SNPs with an outlier test.

Smoking, body mass index, basophil count, and type 2 diabetes as risk factors for COVID-19 may mediate the causal effects of CHB on the three COVID-19 outcomes. Therefore, to calculate the direct causal effect of CHB on COVID-19 and investigate the possible mediating mechanisms of type 2 diabetes, BMI, basophil count, and smoking, MVMR analysis was also performed. The dataset for BMI included 158,284 participants [29], while the dataset for type 2 diabetes comprised 77,418 subjects with the condition and 356,122 control subjects [30]. The smoking behavior data set consisted of 83,810 beginning smokers and 81,626 controls [31]. The basophil count dataset consisted of 62,076 individuals [32]. All of them are of East Asian descent.

In addition, we further evaluated the causal effect of the susceptibility and severity of COVID-19 on CHB in the opposite direction. Due to the limited size of the COVID-19 dataset, we employed inclusive criteria for the identification of significant IVs with a threshold of *P* < 1 × 10^–5^. SNPs were pruned using a pooled r^2^ cutoff of 0.01 within a 500 kb window. Consequently, we performed univariate MR analyses by harmonizing three instrumental variables representing SARS-CoV-2 infection, hospitalized COVID-19, and severe COVID-19 with the CHB dataset.

### Data and resource availability

Summary level data for CHB (bbj-a-99), BMI (bbj-a-1), type 2 diabetes (ebi-a-GCST010118), basophil count (bbj-a-12), and smoking (bbj-a-78) can be acquired from the database of the MRC IEU OpenGWAS Project (https://gwas.mrcieu.ac.uk). The GWAS summary data for three COVID-19 outcomes (all in individuals of East Asian ancestry) can be found on the website of the COVID-19 Host Genetics Initiative (https://www.covid19hg.org/results/r7/).

### Ethics statement

We used publicly available GWAS summary statistics data from Biobank Japan and the COVID-19 Host Genetics Initiative, both of which obtained informed consent from all participants according to their respective institutional review board protocols. As a result, separate ethics approval for this study was unnecessary.

### Statistical analysis

After accounting for multiple testing issues, we used a Bonferroni correction to determine the significance of causal associations with a *P* value < 0.017 (0.05/3 outcomes). The statistical software R (version 4.2.1) was utilized for the analyses, which employed the TwoSampleMR (version 0.5.6) and MRPRESSO (version 1.0) packages.

## Results

### Univariate MR analysis

We obtained 55 independently significant SNPs strongly associated with CHB from the CHB summary statistics of Japan Biobank. The F-statistics for these SNPs ranged from 30 to 168. Since each dataset related to the three COVID-19 outcomes had a distinct set of SNPs, we removed any SNPs that were absent in a particular outcome dataset, resulting in varying sets of IVs for each MR analysis. Specifically, the MR analyses of the associations between CHB and SARS-CoV-2 infection, hospitalized COVID-19, and severe COVID-19 included 53, 53, and 45 IVs, respectively (Additional file 1: Table S2–S4).

**Fig. 1:**
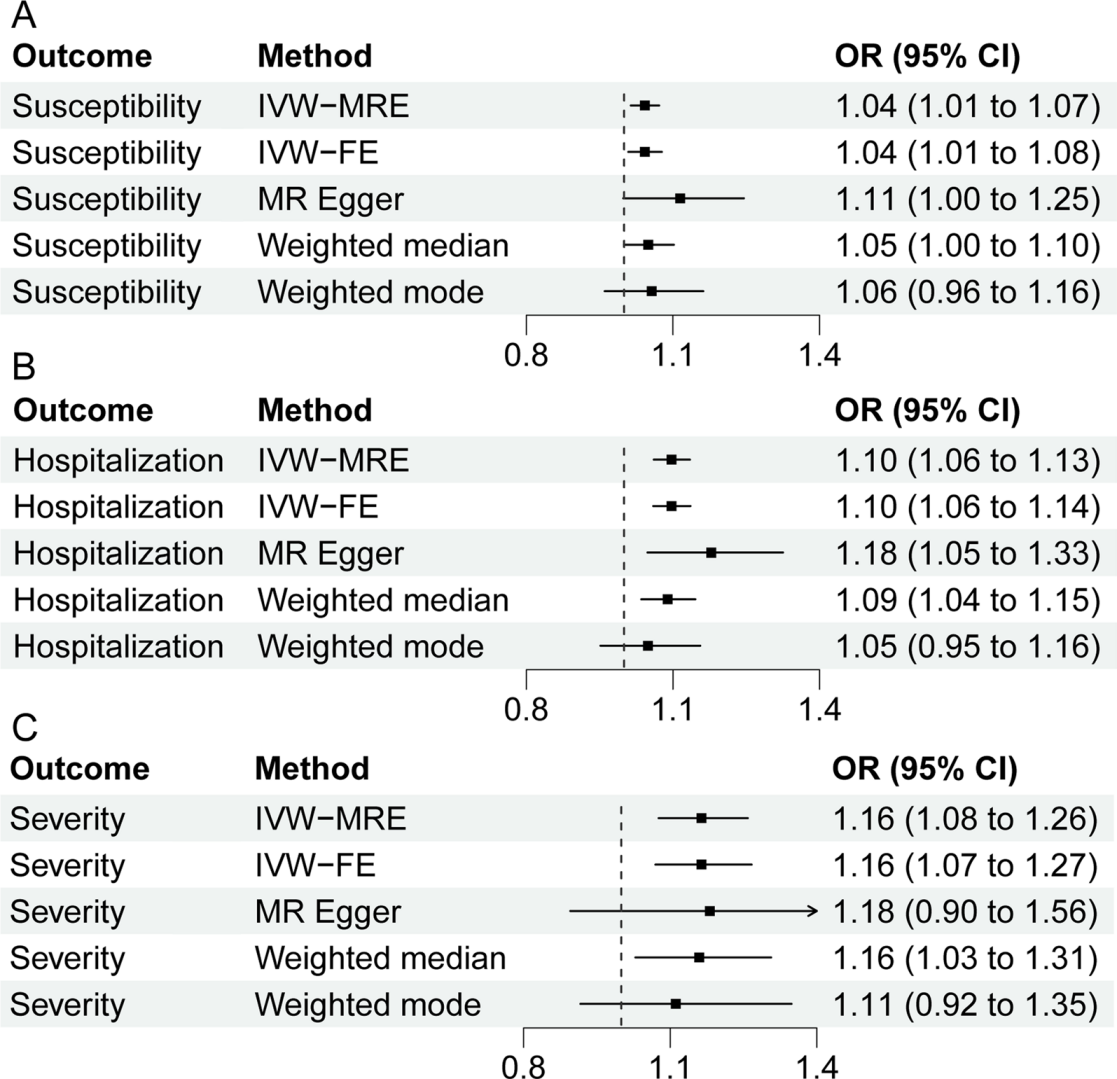
Causal links between CHB and three COVID-19 outcomes in individuals of East Asian ancestry. **A** SARS-CoV-2 infection, **B** hospitalized COVID-19, **C** severe COVID-19. CHB chronic hepatitis B, *IVW-MRE* multiplicative random effects inverse variance weighting, *IVW-FE* fixed effects inverse variance weighting, *OR* odds ratio, *CI* confidence interval

**Fig. 2:**
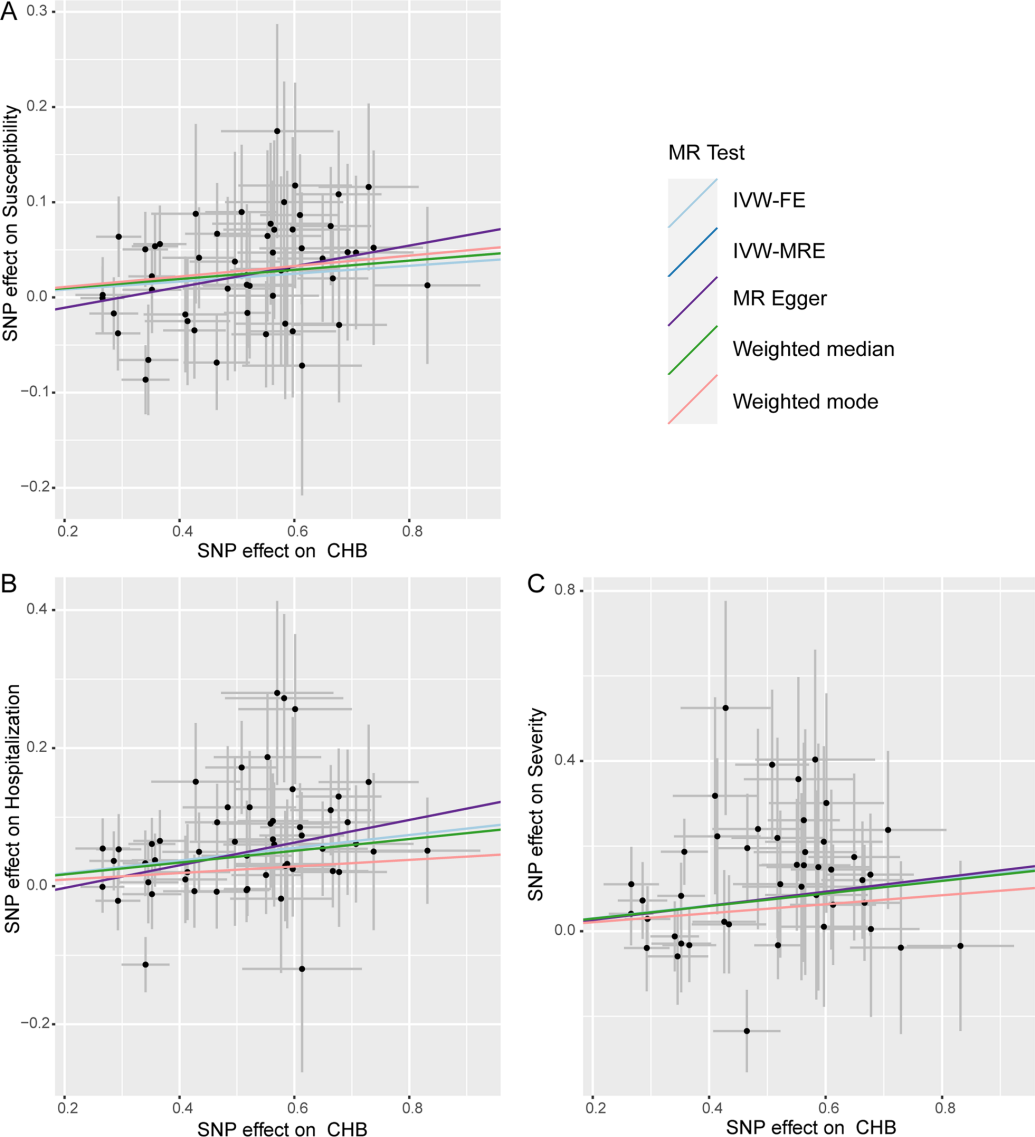
Scatter plots from genetically related CHB on three COVID-19 outcomes. **A** SARS-CoV-2 infection, **B** hospitalized COVID-19, **C** severe COVID-19. *CHB* chronic hepatitis B, *IVW-MRE* multiplicative random effects inverse variance weighting, *IVW-FE* fixed effects inverse variance weighting, *SNP* single nucleotide polymorphism, *MR* Mendelian randomization

Our MR analysis demonstrated that the genetic predisposition to CHB has a significant causal effect on SARS-CoV-2 infection (OR = 1.04, 95% CI 1.01–1.07, *P* = 3.39E−03, IVW-MRE), hospitalized COVID-19 (OR = 1.10, 95%CI 1.06–1.13, *P* = 7.31E−08, IVW-MRE), and severe COVID-19 (OR = 1.16, 95% CI 1.08–1.26, *P* = 1.43E−04, IVW-MRE) (Table 1 and Fig. 1A–C). Moreover, the WM (Weighted median) method also showed that genetically predicted CHB was associated with hospitalized COVID-19 (OR = 1.09, 95% CI 1.04–1.15, *P* = 9.69E−04, WM) and severe COVID-19 (OR = 1.16, 95% CI 1.03–1.31, *P* = 1.52E−02, WM) (Table 1 and Fig. 1B, C). The causal effects obtained by the four methods were in the same direction (Fig. 2).

**Table 1:** Causal effects of CHB on COVID-19 outcomes

Outcome	Method	Beta	SE	OR (95% CI)	*P*
Susceptibility	IVW-MRE	0.042	0.014	1.042 (1.014–1.072)	3.39E−03
Susceptibility	IVW-FE	0.042	0.017	1.042 (1.009–1.077)	1.32E−02
Susceptibility	MR Egger	0.109	0.056	1.115 (0.998–1.245)	5.93E−02
Susceptibility	Weighted median	0.048	0.025	1.049 (1.000–1.102)	5.19E−02
Susceptibility	Weighted mode	0.055	0.049	1.056 (0.960–1.162)	2.64E−01
Hospitalization	IVW-MRE	0.093	0.017	1.097 (1.061–1.135)	7.31E−08
Hospitalization	IVW-FE	0.093	0.018	1.097 (1.060–1.136)	1.51E−07
Hospitalization	MR Egger	0.164	0.060	1.178 (1.048–1.325)	8.39E−03
Hospitalization	Weighted median	0.085	0.026	1.089 (1.035–1.146)	9.69E−04
Hospitalization	Weighted mode	0.048	0.050	1.049 (0.952–1.156)	3.41E−01
Severity	IVW-MRE	0.152	0.040	1.164 (1.076–1.259)	1.43E−04
Severity	IVW-FE	0.152	0.043	1.164 (1.070–1.267)	4.18E−04
Severity	MR Egger	0.166	0.141	1.181 (0.896–1.558)	2.45E−01
Severity	Weighted median	0.148	0.061	1.159 (1.029–1.306)	1.52E−02
Severity	Weighted mode	0.106	0.099	1.112 (0.916–1.348)	2.89E−01

### Sensitivity analyses

**Fig. 3:**
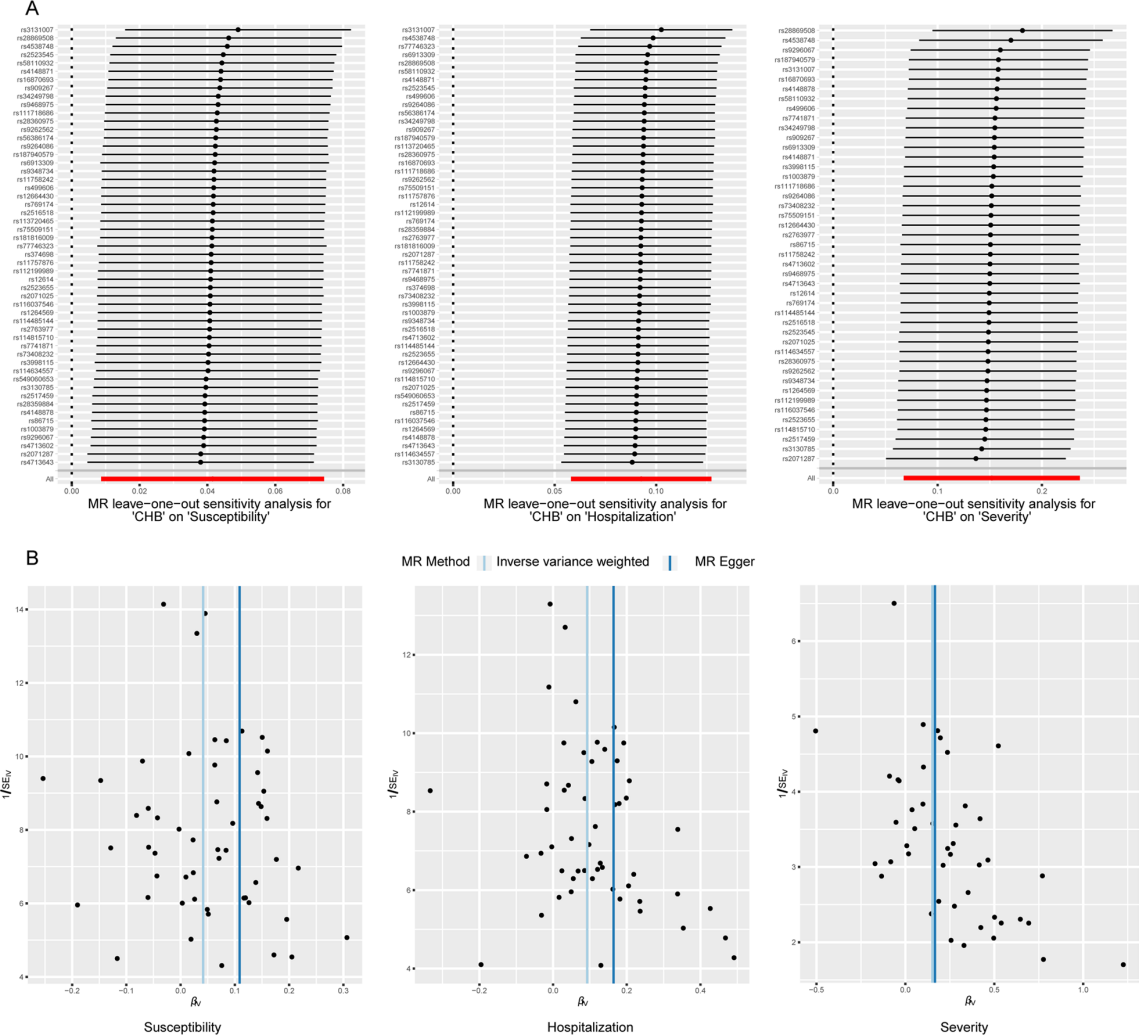
LOO plots and funnel plots from genetically related CHB on COVID-19 outcomes. **A** MR LOO sensitivity analysis for the effect of CHB on COVID-19, **B** Funnel plot of the causal links between genetically related CHB and COVID-19. *LOO* leave-one-out, *CHB* chronic hepatitis B, *MR* Mendelian randomization

We conducted several sensitivity analyses to ensure the robustness of our findings, including the MR-PRESSO global test, the MR-Egger intercept test, and Cochran's Q test. Causal effect estimates were in the same direction for all methods (OR > 1). Neither the MR-PRESSO global test nor the MR-Egger intercept test provided evidence for any pleiotropy (Table 2). The results of the Cochran Q test showed no heterogeneity in the IVs (*P* > 0.05) (Table 2). In addition, no outliers were detected by the MR‐PRESSO method. LOO analyses and funnel plots (Fig. 3A, B) showed that the estimates were not biased by a single SNP, which suggests that the estimates were not violated.

**Table 2:** Sensitivity analysis of the causal association between CHB and the risk of COVID-19

Outcome	Cochran Q test			MR-Egger		MR-PRESSO
	Q value	Q_df	*P*	Intercept	*P*	*P*
Severity	37.8619	44	0.730885	− 0.00681	0.91436	0.761
Hospitalization	49.47599	52	0.573747	− 0.03541	0.216934	0.624
Susceptibility	37.17457	52	0.939859	− 0.03258	0.217301	0.968

### Multivariable MR estimates

The positive associations between genetic susceptibility to CHB and SARS-CoV-2 infection (OR = 1.06, 95% CI 1.02–1.11, *P* = 1.44E-03) and hospitalization for COVID-19 (OR = 1.12, 95% CI 1.07–1.16, *P* = 5.13E−07) persisted even after adjusting for smoking, BMI, basophil count, and type 2 diabetes in the MVMR analysis. However, MVMR was suggestive of a nominally significant causal association between genetically susceptible CHB and severe COVID-19 (OR = 1.12, 95% CI 1.01–1.23, *P* = 0.029) (Table 3). This result raises the possibility that smoking, type 2 diabetes, basophil count, and BMI might mediate the link between CHB and severe COVID-19.

**Table 3:** MVMR analysis between CHB and the COVID-19 outcomes

Exposure	Outcome	BETA	SE	OR (95% CI)	*P*
BMI	Susceptibility	0.381	0.115	1.46 (1.17–1.84)	9.64E−04
Basophil count	Susceptibility	0.092	0.096	1.10 (0.91–1.32)	3.42E−01
Smoking behaviors	Susceptibility	2.92	0.834	18.55 (3.62–95.03)	4.59E−04
CHB	Susceptibility	0.063	0.02	1.06 (1.02–1.11)	1.44E−03
Type 2 diabetes	Susceptibility	0.079	0.034	1.08 (1.01–1.16)	2.12E−02
BMI	Hospitalization	0.679	0.135	1.97 (1.51–2.57)	4.91E−07
Basophil count	Hospitalization	0.008	0.114	1.01 (0.81–1.26)	9.47E−01
Smoking behaviors	Hospitalization	2.383	0.987	10.84 (1.57–74.98)	1.57E−02
CHB	Hospitalization	0.11	0.022	1.12 (1.07–1.16)	5.13E−07
Type 2 diabetes	Hospitalization	0.141	0.041	1.15 (1.06–1.25)	5.77E−04
BMI	Severity	1.091	0.27	2.98 (1.75–5.06)	5.45E−05
Basophil count	Severity	− 0.372	0.226	0.69 (0.44–1.07)	1.00E−01
Smoking behaviors	Severity	− 0.337	1.996	0.71 (0.01–35.71)	8.66E−01
CHB	Severity	0.11	0.05	1.12 (1.01–1.23)	2.90E−02
Type 2 diabetes	Severity	0.234	0.081	1.26 (1.08–1.48)	3.67E−03

### Reverse MR analysis

**Fig. 4:**
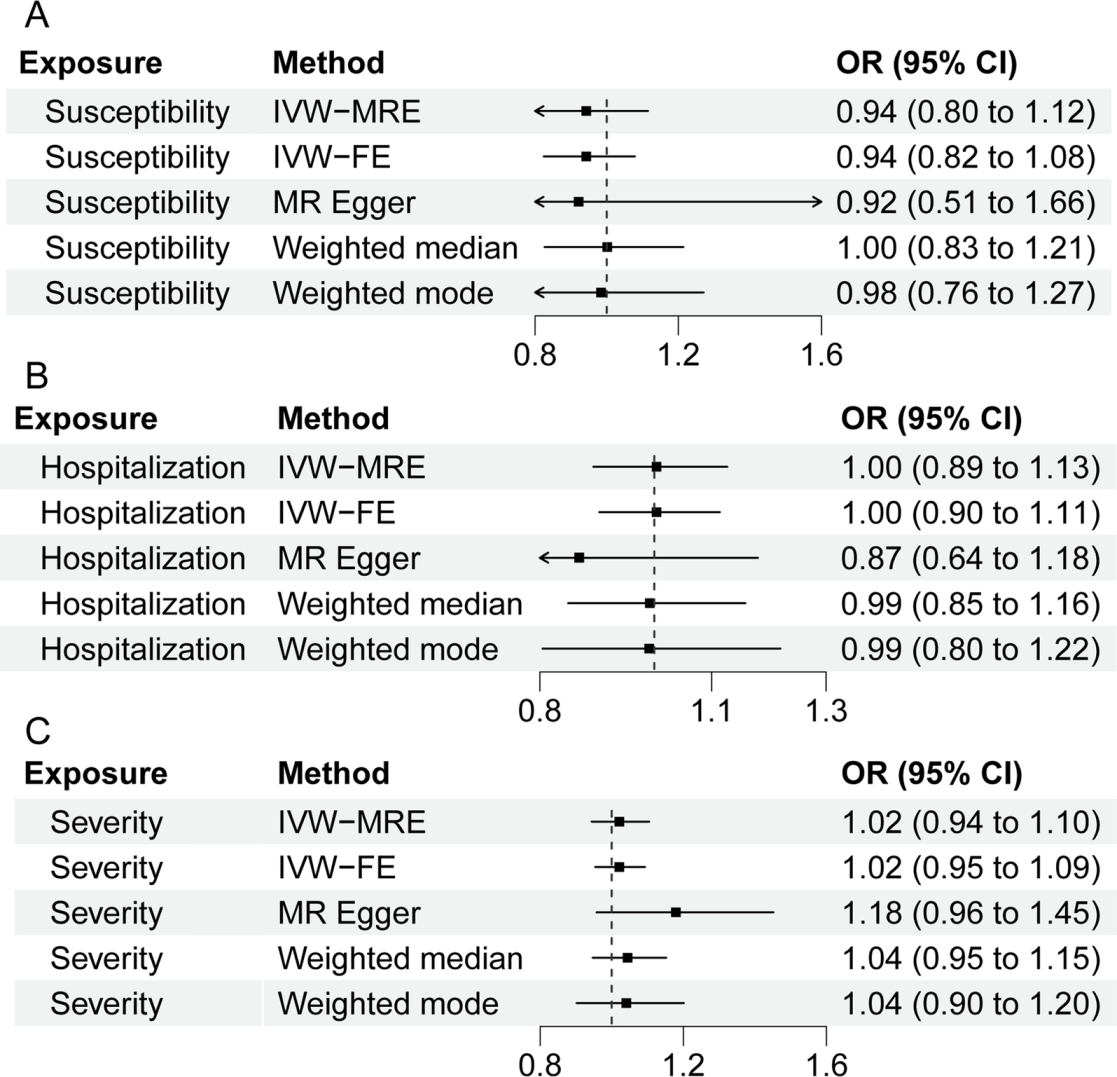
Causal effects of the COVID‐19 outcomes on CHB in individuals of East Asian ancestry. **A** SARS-CoV-2 infection, **B** hospitalized COVID-19, **C** severe COVID-19. *CHB* chronic hepatitis B, *IVW-MRE* multiplicative random effects inverse variance weighting, *IVW-FE* fixed effects inverse variance weighting, *OR* odds ratio, *CI* confidence interval

We further explored the causal effects of susceptibility and severity of COVID-19 on CHB. Thirteen SNPs were used to represent susceptibility to COVID-19, 17 SNPs for hospitalized COVID-19, and 13 SNPs for severe COVID-19. Inverse MR analysis revealed no apparent causal association between genetically predicted susceptibility and severity of COVID-19 and CHB (Fig. 4A–C). Sensitivity analysis confirmed the reliability of the results (Additional file 1: Table S5).

## Discussion

The potential causal links between CHB and COVID-19 susceptibility and severity are being investigated for the first time utilizing MR in this investigation. Univariate MR analysis supported that genetically susceptible CHB increased the risk of COVID-19 infection and exacerbation in the East Asian population. We found that the direct causal effect of CHB on SARS-CoV-2 infection and hospitalized COVID-19 was not affected by potential confounding factors such as smoking behavior, BMI, basophil count, and type 2 diabetes, suggesting that these comorbidities may not play a mediating role. Nevertheless, after accounting for these comorbidities, the direct causative effect of CHB on severe COVID-19 was partially attenuated. In addition, we found no causal effect of COVID-19 on CHB.

Regarding CHB's susceptibility to COVID-19, some clinical investigations from China have discovered that HBV infection rates in the general Chinese population are significantly greater than those in COVID-19 patients [18, 33–36]. According to Kang et al. [37], SARS-CoV-2 testing for CHB in the Korean population exhibited a low positive rate. Similarly, individuals with cirrhosis in North America had a decreased likelihood of contracting SARS-CoV-2 infection [38], which may be attributed to increased testing, greater attention, and improved patient compliance with public health recommendations. In addition, several studies have shown that antiviral drugs, such as tenofovir and entecavir, may reduce susceptibility to COVID-19 [37, 39]. Meanwhile, in a retrospective study of 19,160 COVID-19 patients from South Korea, antiviral therapy in HBV-infected patients was not associated with lower death rates, hospitalization, or ICU admission compared with those who did not receive antiviral therapy [40]. our study showed that both univariate and multivariate MR analyses suggested that CHB increased the susceptibility to COVID-19.

Some evidence suggests that SARS-CoV-2 is present in the liver of severe COVID-19 patients [41, 42]. The liver is impacted by SARS-CoV-2 infection, according to earlier clinical reports [4, 33, 43]. Direct viral effects on the cell, vascular damage and coagulopathy, excessive systemic immune response, and drug-induced hepatotoxicity are possible causes of liver damage during SARS-CoV-2 infection [44]. Most data from retrospective studies suggest that cirrhosis, alcohol-related liver disease, or non-alcoholic fatty liver disease are danger signals for COVID-19 exacerbations [45]. However, there is significant controversy as to whether CHB affects the severity of COVID-19.

According to some studies, COVID-19-positive CHB patients have a higher risk of developing a severe illness and a worse prognosis. Chen and colleagues discovered that nearly half (47%) of HBV patients with COVID-19 were defined as severe cases [21]. Similarly, a multi-site historical analysis found that the proportion of severe cases in CHB patients [27.52%, (30/109)] was markedly above that in non-CHB patients [5.20%, (17/327)] [46]. A single-center study from Wuhan showed that COVID-19 patients co-infected with HBV during the HBeAg-positive CHB/infection phase were more likely to be admitted to ICU and die [47]. However, other studies do not support these conclusions. A Chinese retrospective study involving 347 patients showed no difference in the probability of severe COVID-19 or period for clearance of SARS-CoV-2 between patients with and without HBV [47]. Liu et al. [48] found that 21 patients with both COVID-19 and chronic HBV infection had neither prolonged clearance of SARS-CoV-2 nor exacerbated COVID-19 progression compared with 51 patients with COVID-19 and absence of HBV infection. The relatively small sample sizes and nonrandomized controls included in these studies may have affected the accuracy of the results. MVMR analysis, after adjusting for smoking behavior, type 2 diabetes, basophil count, and BMI, further revealed that CHB directly raised the risk of hospitalized COVID-19. Whereas, the causal relationship between CHB and severe COVID-19 was partially mediated by smoking, type 2 diabetes, basophil count, and BMI.

This study has several strengths, including the use of MR analysis. The inclusion of participants with the same East Asian ancestry in the GWAS datasets also reduces potential population heterogeneity. Sensitivity analyses were conducted to ensure the validity of the results, and both the total and direct effects of genetically related CHB on the three COVID-19 outcomes were investigated. However, there are limitations to this study, including the relatively small sample size of the GWAS data sets for COVID-19, particularly severe cases. The findings may also not be generalizable to other ancestral populations, given that the analysis was performed only on individuals of East Asian ancestry. Additionally, other confounding factors besides smoking, BMI, basophil count, and type 2 diabetes may mediate the observed causal relationships.

## Conclusions

This study is the first to provide robust evidence of the causal relationship between CHB and both susceptibility and severity of COVID-19 through the application of MR analysis.

## Supplementary Information

**Additional file 1**. **Table 1**. Instrumental variables strongly associated with CHB. **Table 2**. Detailed data after harmonization of instrumental variables for CHB with the SARS-CoV-2 infection dataset. **Table 3**. Detailed data after harmonization of instrumental variables for CHB with the hospitalized COVID-19 dataset. **Table 4**. Detailed data after harmonization of instrumental variables for CHB with the severe COVID-19 dataset. **Table 5**. Sensitivity analysis of the causal association of COVID-19 with CHB.

## Data Availability

Summary level data for CHB (bbj-a-99), BMI (bbj-a-1), type 2 diabetes (ebi-a-GCST010118), basophil count (bbj-a-12), and smoking (bbj-a-78) can be acquired from the database of the MRC IEU OpenGWAS Project (https://gwas.mrcieu.ac.uk). The GWAS summary data for three COVID-19 outcomes (all in individuals of East Asian ancestry) can be found on the website of the COVID-19 Host Genetics Initiative (https://www.covid19hg.org/results/r7/).

## Abbreviations

BBJ: BioBank Japan
BMI: Body mass index
CHB: Chronic hepatitis B
COVID-19: Coronavirus disease 2019
HBV: Hepatitis B virus
IV: Instrumental variant
IVW-FE: Fixed effects inverse variance weighting
IVW-MRE: Multiplicative random effects inverse variance weighting
LOO: Leave-one-out
MR: Mendelian randomization
SNPs: Single nucleotide polymorphisms
SARS-CoV-2: Severe acute respiratory syndrome coronavirus 2
WM: Weighted median
